# Pharmacokinetics and Tissue Distribution of Enrofloxacin Following Single Oral Administration in Yellow River Carp (*Cyprinus carpio haematoperus*)

**DOI:** 10.3389/fvets.2022.822032

**Published:** 2022-02-04

**Authors:** Fan Yang, Chao-Shuo Zhang, Ming-Hui Duan, Han Wang, Zhe-Wen Song, Hao-Tian Shao, Kai-Li Ma, Fang Yang

**Affiliations:** Department of Veterinary Pharmacology, College of Animal Science and Technology, Henan University of Science and Technology, Luoyang, China

**Keywords:** enrofloxacin, Yellow River carp, pharmacokinetics, tissue distribution, oral administration

## Abstract

The pharmacokinetics and tissue distribution of enrofloxacin were determined in Yellow River carp (*Cyprinus carpio haematopterus*) reared at 20°C after single oral administration of enrofloxacin at 10 mg·kg^−1^ body weight (BW). Plasma, bile, and different tissue samples, including liver, kidney, gill, gut, and skin-muscle, were collected at predetermined times points. An HPLC method was developed to simultaneously determine the concentrations of enrofloxacin and its metabolite, ciprofloxacin. However, ciprofloxacin was only detectable in some liver samples with trace levels. Then the average enrofloxacin concentrations vs. time data were subjected to a non-compartmental analysis using WinNonLin 5.2 software. Multiple peaking profiles were observed in all enrofloxacin concentration-time curves. The peak concentration (C_max_) values were observed as 0.79, 1.01, 2.09, 2.85, 4.34, 10.78, and 13.07 μg·ml^−1^ (or g^−1^) in plasma, skin-muscle, gill, kidney, liver, bile, and gut, respectively, and the corresponding time to reach peak concentration (T_max_) was 8, 8, 1, 8, 1, 72, and 4 h, respectively. The values of elimination half-life (T_1/2λ*Z*_) of enrofloxacin in different tissues was in the following order: gill (291.13 h) > liver (222.29 h) > kidney (157.22 h) > plasma (129.44 h) > gut (91.47 h) > skin-muscle (87.77 h) > bile (86.22 h). The present results showed that enrofloxacin had a wide distribution in different tissues, however slow absorption and elimination in Yellow River carp. Additionally, enrofloxacin exhibited large distribution in bile, indicating that bile excretion might be the primary elimination route of enrofloxacin in Yellow River carp. A withdrawal period was calculated as 379.2 °C-day for single oral dosing of enrofloxacin at 10 mg/kg BW. Based on the calculated PK/PD indices of AUC/MIC or C_max_/MIC, the current enrofloxacin dosing regimen might have a positive therapeutic effect on the infection of *Flavobacterium columnare, Aeromonas sobria*, or *Aeromonas hydrophila*. However, the depletion study following multiple oral doses should be carried out in Yellow River carp reared at lower temperatures, and the withdrawal period should also be further calculated.

## Introduction

Common carp (*Cyprinus carpio*), with the characteristics of strong adaptability, excellent reproductive ability, and rich nutritional value, has been reared in Asia for more than 2 000 years (1). Especially in China, carp has become one of the most commonly farmed fish species. Yellow River carp (*Cyprinus carpio haematoperus*) mainly refers to the carp living in the Yellow River (2). However, with the development of freshwater aquaculture, Yellow River carp have also been cultivated artificially in a wide temperature range of 5–28°C, and it has stronger disease resistance and more delicious meat (3).

With the rapid development of intensive aquaculture, fish suffer from many pathogens (4). Lepidorthosis is a common disease in the Yellow River carp, which has caused substantial economic, and its pathogenic bacteria included *Aeromonas hydrophila* and *Aeromonas sobria* (5). Columnaris disease is another common disease in the Yellow River carp caused by *Flavobacterium columnare* (6). To reduce these losses, treatment with effective antibacterial agents is necessary.

Enrofloxacin is a fluoroquinolone antibiotic that inhibits bacterial DNA-gyrase (7). Because of its broad antimicrobial spectrum, enrofloxacin has been licensed for use in bovine, swine, poultry, and aquaculture species (8). Enrofloxacin serves as a last-line treatment in aquaculture, and it effectively treats the bacterial infections caused by *F. columnare* (6), *A. hydrophila* (9), and *A. sobria* (10). The plasma pharmacokinetics of enrofloxacin has been reported in some fish species, such as crucian carp (11, 12), large yellow croaker (13), tilapia (14), snakehead fish (15), brown turbot (16), and salmonids (17). In addition to those pharmacokinetics studies, the tissue distribution of enrofloxacin has also been reported in aquaculture species, including northern snakeheads (18), rainbow trout (19, 20), grass carp (21), largemouth bass (22), and pacu (23). Those previous results indicated that the plasma and tissues kinetics of enrofloxacin might be strongly influenced by fish species, body conditions, routes of administration, water temperature, and other environmental factors. However, neither the pharmacokinetics nor tissue distribution is known for enrofloxacin in the Yellow River carp. Therefore, this study aimed to investigate enrofloxacin's pharmacokinetics and tissue distribution and its primary metabolite, ciprofloxacin, in Yellow River carp after single oral dosing.

## Materials and Methods

### Chemical Reagents

The analytical standards of enrofloxacin (Lot No. H0081907) and ciprofloxacin hydrochloride (Lot No. H0101310) were purchased from the China Institute of Veterinary Drugs Control (Beijing, China), with the purities at 99.7 and 99.8%, respectively. Enrofloxacin hydrochloride soluble powder (30%; Lot No. 21011602) was obtained from Luoyang Xianger Biological Technology Co., Ltd. (Luoyang, China). Acetonitrile, dichloromethane, and hexane were HPLC grade and purchased from Tianjin Kemi O Chemical Reagent Co., Ltd. (Tianjin, China).

### Animals

Hundred healthy Yellow River carp (*Cyprinus carpio haematoperus*) were purchased from Mianchi Qinglianhe Aquaculture Co., Ltd. (Sanmenxia, China). Their average body weight (BW) was 0.41 kg (0.29–0.64 kg). Eighty-five fish in the population were equally and randomly divided into 17 groups, and the other 15 fish served as a control group to supply blank plasma and tissues samples. Each treatment group (*n* = 5) was reared in a cuboid tank (1.3 × 0.8 × 0.65 m, length × width × height) under continuous aeration. The water was analyzed daily for quality control. The pH was approximately 7.3, and the dissolved oxygen and ammonia concentrations were >8 and about 0.1 mg/L, respectively. The water temperature was kept at 20 ± 0.8°C with heat rods. All fishes were allowed to acclimate for at least seven days and fed daily with a drug-free dry feed (pellet size 3 mm) purchased from Henan Tongwei Feed Co., Ltd. (Xinxiang, Henan, China). Animal experiments were conducted under protocols approved by the Institutional Animal Care and Use Committee (IACUC) of Henan University of Science and Technology (approved # 20201003).

### Drug Administration and Sampling

Enrofloxacin solution (10 mg·ml^−1^, calculated as pure enrofloxacin) was prepared by dissolving enrofloxacin hydrochloride soluble powder in deionized water. This solution was mixed with drug-free feed to obtain a suspension with a final enrofloxacin concentration of 5 mg·g^−1^, thoroughly mixed and prepared at least 6 h before administration. As described previously (24), a 1 ml syringe attached to a 96 mm-long stainless-steel gavage needle was used to administer enrofloxacin by oral gavage at the dose of 10 mg·kg^−1^ BW. Oral administration was carried out without the use of any anesthetic. After oral administration, each fish was manually restrained vertically for approximately 20 s and then transferred to a single tank to check for possible regurgitation for about 3 min. No regurgitation was observed for any fish.

Following enrofloxacin dosing, five fish in one group were randomly collected blood samples through the fishtail vein, then sacrificed by a blow to the head. And samples of bile, liver, kidney, gill, gut, and muscle plus skin were collected at 5, 15, 30 min, 1, 2, 4, 6, 8, 12, 24, 72, 96, 120, 144, 240, 360, and 480 h. When collecting intestinal samples, the intestinal contents were excluded by squeezing rather than flushing. And only gut tissue was collected. Plasma samples were further collected by centrifugation at 2,000 × g for 10 min. All collected samples were frozen and stored at −20°C until further analysis.

### Analytical Method

Enrofloxacin concentrations and its metabolite, ciprofloxacin, were determined using a previous HPLC method (25). Briefly, 0.2 ml of plasma sample was mixed with 0.4 ml of acetonitrile, followed by vortexing for 30 s and centrifugation at 8,000 × g for 10 min; the supernatant was transferred to a clean glass tube, and the sediment was re-extracted. All supernatants were collected and evaporated to dryness with a stream of nitrogen at 45°C. The residue was redissolved in 0.5 ml of the mobile phase. After vortexing for 1 min and centrifugation for 10 min at 15,000 × g, the supernatant (20 μl) was injected onto the C-18 column (Hypersil BDS C18; 250 × 4.6 mm inner diameter, 5 μm; Dalian Elite Analytical Instruments Co., Ltd.). The tissue samples were firstly homogenized using an A300-36G tissue homogenizer (IKA, Guangzhou, China), then acidified acetonitrile (100 ml of acetonitrile was mixed with 0.8 mL of 50% hydrochloric acid) was used to extract enrofloxacin and ciprofloxacin. One volume of tissue (1 g for muscle plus skin and 0.5 g for the other tissues) was mixed with three volumes of acidified acetonitrile. After vortexing, centrifuging, mixing, and drying, 0.5 ml of mobile phase was used to dissolve the residue, and 1 ml of hexane was added to the solution, followed by complete vortexing and centrifugation at 8,000 × g for 10 min. The below layer (20 μl) was injected onto the C-18 column. For bile samples, the acidified acetonitrile was changed to dichloromethane, and the other operations were consistent with the tissues samples.

The Waters e2695 HPLC system (Waters, USA) was used to determine the concentrations of enrofloxacin and ciprofloxacin with a 2475 fluorescence detector. The chromatographic column was Hypersil BDS C18 (250 × 4.6 mm inner diameter, 5 μm, Dalian Elite Analytical Instruments Co., Ltd.) kept at 30°C. The mobile phase was 18 % acetonitrile and 82% phosphoric acid buffer (0.05%; adjusting the pH to 2.8 with triethylamine). And its flow rate was set as 1 ml/min. The excitation and emission wavelengths of the fluorescence detector were 280 and 450 nm, respectively.

### Data Analysis

Following single oral administration of enrofloxacin, ciprofloxacin was only detected in some samples at trace levels (data not shown here). Therefore, the ciprofloxacin kinetics were not determined in this study. The average concentrations of enrofloxacin at each time point were calculated. Then mean concentrations vs. time data were subjected to non-compartmental analysis (26) by WinNonlin (Version 5.2; Pharsight Corporation, Mountain View, CA, USA). The area under the concentration-time curve (AUC_0−∞_) and the first-moment curve (AUMC_0−∞_) were calculated using the linear trapezoidal method (27). The peak concentration of enrofloxacin (C_max_) and time to reach it (T_max_) were both directly read from the average concentration vs. time curve. The elimination rate constant (λ_Z_) was estimated by linear regression of mean drug concentrations vs. time data. And the elimination half-life (T_1/2λ*Z*_) was calculated as 0.693/λ_Z_. Mean residence time (MRT) was calculated as the ratio of AUMC_0−∞_ to AUC_0−∞_. All these parameters were determined for all collected samples. The withdrawal period was calculated by linear regression analysis of enrofloxacin concentrations in skin-muscle through the software of WT 1.4 (23). And it was determined at the time when the 95% upper one-sided tolerance limit was below the maximum residue limit in skin-muscle (100 μg/kg) with 95% confidence.

## Results

### HPLC Method

The present HPLC method was found to be linear and reproducible for both enrofloxacin and ciprofloxacin in tissues and body fluid in concentrations ranging from 0.01 to 15 μg/g or μg/ml. The limits of detection (LOD) and quantification (LOQ), based on a signal-to-noise ratio >3 and >10, for both enrofloxacin and ciprofloxacin were 0.005 and 0.01 μg/ml (or μg/g), respectively, in all collected samples. In order to monitor the precision and accuracy of this assay, three replicates of enrofloxacin and ciprofloxacin at different spiked concentrations were tested 3 consecutive days to evaluate the coefficients of variation and recovery rates. The results showed that all inter- and intra-day coefficients of variation were below 4.78% for enrofloxacin and below 5.79% for ciprofloxacin. The average recovery rates were above 86.72 and 89.95% for enrofloxacin and ciprofloxacin, respectively. The detailed HPLC results are shown in Table 1.

**Table 1:** The results of the analytical method for the determination of enrofloxacin and ciprofloxacin concentrations in fish tissues and body fluid.

**Tissues or body fluid**	**Enrofloxacin**				**Ciprofloxacin**			
	**Spiked concentrations (μg/g or μg/ml)**	**Recovery rate (%, Mean ±SD)**	**Inter-day coefficients (%, range)**	**Intra-day coefficients (%)**	**Spiked concentrations (μg/g or μg/ml)**	**Recovery rate (%, range)**	**Inter-day coefficients (%, range)**	**Intra-day coefficients (%)**
Plasma	0.01	90.89 ± 2.78	1.51–4.12	3.06	0.01	93.96 ± 2.63	1.46–3.64	2.79
	0.5	87.88 ± 2.39	2.25–3.85	2.72	0.5	91.05 ± 2.18	2.18–3.45	2.39
	5	86.72 ± 1.76	1.61–2.92	2.03	5	89.95 ± 1.54	0.91–2.55	1.71
Bile	0.01	94.33 ± 2.59	2.03–3.14	2.75	0.01	95.31 ± 2.62	2.62–3.79	2.75
	0.5	94.98 ± 3.63	1.15–5.49	3.82	0.5	93.51 ± 3.18	3.60–3.77	3.41
	15	91.93 ± 3.80	3.40–4.18	4.13	15	93.42 ± 3.93	3.29–4.99	4.20
Skin–muscle	0.01	91.47 ± 4.23	2.52–4.10	4.63	0.01	93.11 ± 2.28	2.43–2.76	2.45
	0.5	89.78 ± 3.19	1.00–4.23	3.55	0.5	90.84 ± 3.34	1.37–5.79	3.68
	5	91.04 ± 3.90	2.01–4.23	4.29	5	91.02 ± 2.04	2.12–2.98	2.24
Liver	0.01	90.77 ± 3.15	2.29–3.92	3.47	0.01	92.73 ± 3.35	2.01–5.27	3.61
	0.5	88.52 ± 3.06	2.46–4.78	3.46	0.5	91.56 ± 2.30	2.30–3.47	2.52
	5	88.58 ± 2.50	1.89–3.61	2.83	5	92.45 ± 3.27	2.30–4.36	3.53
Kidney	0.01	95.60 ± 3.96	3.00–4.62	4.14	0.01	91.57 ± 2.33	2.73–2.92	2.55
	0.5	92.14 ± 2.93	1.15–4.48	3.18	0.5	92.43 ± 2.57	2.53–3.53	2.78
	5	90.93 ± 3.09	1.27–4.34	3.40	5	91.98 ± 2.14	1.37–3.43	2.33
Gill	0.01	95.89 ± 2.78	1.43–3.90	2.90	0.01	94.05 ± 3.13	1.32–4.76	3.33
	0.5	89.62 ± 3.26	3.28–4.49	3.64	0.5	91.42 ± 2.10	1.89–2.81	2.29
	5	89.10 ± 2.93	1.71–3.51	3.28	5	90.97 ± 2.14	1.08–3.17	2.36
Gut	0.01	96.03 ± 2.98	2.72–3.62	3.10	0.01	94.02 ± 2.60	2.52–2.85	2.77
	0.5	87.67 ± 2.64	2.11–4.29	3.01	0.5	95.49 ± 2.33	2.41–3.28	2.44
	15	92.15 ± 4.17	2.69–4.25	4.52	15	92.57 ± 3.26	2.34–4.10	3.52

### Plasma Pharmacokinetics

**Figure 1:**
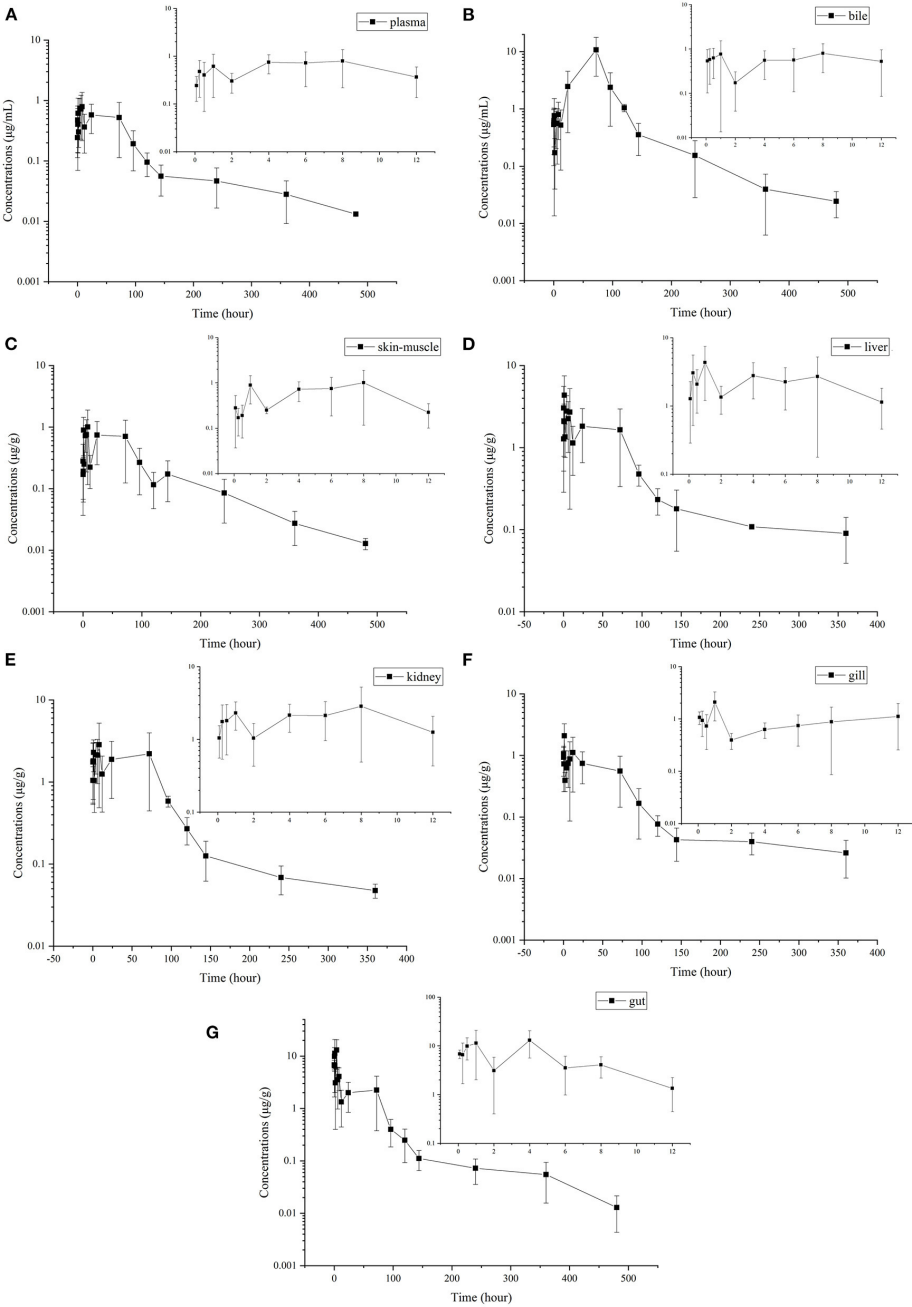
Mean (±SD) concentrations (μg·mL^−1^ or μg·g^−1^) of enrofloxacin following single oral administration of enrofloxacin at 10 mg/kg BW in Yellow River carp (*Cyprinus carpio haematoperus*) reared at 20°C [**(A)** for plasma, **(B)** for bile, **(C)** for skin-on muscle, **(D)** for liver, **(E)** for kidney, **(F)** for gill, and **(G)** for gut]. All five samples at the last sampling point could be quantified in bile, gill, and gut. However, in plasma, kidney, skin-muscle, and liver, only 2, 2, 2, and 4 samples could be quantified at the last quantifiable time point, respectively (their concentrations were greater than the LOQ). Those samples below the LOQ were omitted and not used to calculate pharmacokinetic parameters and withdrawal period.

Enrofloxacin was detected in plasma, bile, and tissue samples following single oral administration. However, ciprofloxacin was only detectable in a few liver samples with concentrations between LOD and LOQ (data not shown here). Therefore, only enrofloxacin concentration data are presented in Figure 1. Enrofloxacin was detectable for 480 h in plasma, bile, gut, and muscle plus skin, whereas it was detected within 360 h in the liver, kidney, and gill. The multiple-peak phenomenon was found in all samples. The first peak concentrations were generally found around 8 h, and the last peak was observed between 24 and 72 h.

The pharmacokinetic parameters were calculated based on the average concentrations vs. time data in plasma (Table 2). The highest plasma concentration (0.79 μg/ml) was observed at 8 h following oral administration of enrofloxacin. And a T_1/2λ*Z*_ was calculated to be 129.44 h.

**Table 2:** Pharmacokinetics parameters of enrofloxacin in plasma, bile, and different tissues of Yellow River carp (*Cyprinus carpio haematoperus*) after oral administration of single-dose (10 mg/kg BW) at 20°C.

**Parameters**	**Unit**	**Plasma**	**Bile**	**Skin-muscle**	**Liver**	**Kidney**	**Gill**	**Gut**
λ_Z_	h^−1^	0.0054	0.008	0.0079	0.0031	0.0044	0.0024	0.0076
T_1/2λ*Z*_	h	129.44	86.22	87.77	222.29	157.22	291.13	91.47
T_max_	h	8	72	8	1	8	1	4
C_max_	μg·ml^−1^ or μg·g^−1^	0.79	10.78	1.01	4.34	2.85	2.09	13.07
AUC_0−∞_	h·μg·ml^−1^ or h·μg·g^−1^	67.45	597.07	90.99	220.31	216.18	85.19	255.91
AUMC_0−∞_	h^2^·μg·ml^−1^ or h^2^·μg·g^−1^	7524.52	48284.48	9545.86	32467.22	19415.37	13610.36	16094.27
MRT	h	111.56	80.87	104.91	147.37	89.81	159.77	62.89
AUC_%Extrap_	%	3.7521	0.4078	1.6786	12.39	4.7852	13.40	0.763
V_d_/F	ml/kg	27687.4	NA	NA	NA	NA	NA	NA
Cl/F	ml/h/kg	148.3	NA	NA	NA	NA	NA	NA

*λ_Z_, the elimination rate constant; T_1/2λZ_, the elimination half-life; T_max_, the time to reach peak concentration; C_max_, the peak concentration of enrofloxacin; AUC_0−∞_, the area under the concentration-time curve; AUMC_0−∞_, the area under the first-moment curve; MRT, mean residence time; AUC_%Extrap_, percentage of AUC_0−∞_ due to extrapolation from the last time point to infinity; V_d_/F, volume of distribution; Cl/F, clearance; NA, not applicable*.

### Tissue Distribution

The concentration-time profiles of enrofloxacin in different tissues are also shown in Figure 1. The multiple-peak phenomenon was observed in all collected samples; however, only in bile, the last peak concentration was higher than those previous peaks (Figure 1B). The kinetic parameters were also calculated in different tissues and are shown in Table 2. The values of T_1/2λ*Z*_ in different tissues was in the following order: gill (291.13 h) > liver (222.29 h) > kidney (157.22 h) > plasma (129.44 h) > gut (91.47 h) > skin-muscle (87.77 h) > bile (86.22 h). Following single oral administration of enrofloxacin at 10 mg/kg BW, the highest enrofloxacin concentration was measured in the gut (13.07 μg/g) at 4 h, followed by bile (10.78 μg/ml) at 72 h. The peak concentrations of enrofloxacin in all tissues, ranging from 1.01 to 13.07 μg/g, were higher than plasma (0.79 μg/ml), indicating a wide distribution of enrofloxacin in tissues. The AUC_0−∞_ values of enrofloxacin were determined as 597.07, 255.91, 220.31, 216.18, 90.99, 85.19, and 67.45 h·μg/g (or ml) in bile, gut, liver, kidney, skin-muscle, gill, and plasma, respectively. Following the single oral administration of enrofloxacin, the withdrawal period was calculated as 18.96 days. Change its unit from day to °C–day, and its value was 379.2.

## Discussion

To our knowledge, the pharmacokinetics and tissue distribution of enrofloxacin in Yellow River carp were determined here for the first time. Following single oral administration of enrofloxacin at 10 mg/kg BW, ciprofloxacin, the metabolite of enrofloxacin, was only detected in some liver samples with trace levels. Ciprofloxacin with low concentrations has also been reported in the other fish species (11, 13, 18, 28, 29), and it was not detected in *Takifugu flavidus* (30), rainbow trout (17, 19), European cuttlefish (31), Atlantic salmon (32), and crucian carp (33). These results indicated a low metabolization into ciprofloxacin for enrofloxacin in fish species. It is well known that enrofloxacin would mainly be N-dealkylated to ciprofloxacin in animals (7). However, in different species, the metabolic capacity varied greatly. Based on the ratio for the AUC of ciprofloxacin and enrofloxacin, the metabolite concentration was approximately 1.8, 2, 5, and 6% in turbot (34), crucian carp (11), Nile tilapia (14), and red pacu (29), respectively. While the AUC ratio between ciprofloxacin and enrofloxacin was high up to 20, 51.5, and 55% in donkeys (35), pigs (36), and sheep (37), respectively. These results indicated that the extent of demethylation of enrofloxacin to ciprofloxacin was low in many fish species compared with homoiothermic species. Previous studies have proved that enrofloxacin was a potent inhibitor of the drug-metabolizing system in fish species (38, 39); thus, it was probable that enrofloxacin also inhibited its demethylation in Yellow River carp. The *in vivo* or *in vitro* metabolism experiment should be further carried out to validate this hypothesis.

After single oral administration, the average peak concentration in plasma was observed as 0.79 μg/ml at 8 h, indicating a slow absorption. When normalized by the administered doses, the current C_max_ was close to that reported in rainbow trout reared at 16.3°C [0.079 vs. 0.09; (19)]; however, lower than those reported in trout [0.0886–0.189; (40)], tilapia [0.124; (14)], Atlantic salmon [0.154; (41)], red pacu [0.16; (29)], crucian carp [0.266–0.355; (12)], large yellow croaker [0.35; (13)], European cuttlefish [1.095; (31)], and Koi carp [1.436; (42)]. However, the T_max_ value was close to those in Atlantic salmon [6 h; (41)], trout [6–8 h; (40)], large yellow croaker [7.7–8.1 h; (13)], and tilapia [8 h; (14)], longer than those in Koi carp [0.25 h; (42)], crucian carp [0.5–0.75 h; (12)], and European cuttlefish [1 h; (31)], and shorter than that in red pacu [36 h; (29)]. Many factors could affect the drug absorption in fish species, such as water temperature, salinity, eating habits, infection, and sampling design. The present results showed that the absorption of enrofloxacin was slow in Yellow River carp. The oral bioavailability was reported as 86% in crucian carp (33), indicating a complete absorption for enrofloxacin. However, similar results in Yellow River carp need further experimental verification. Additionally, considering the reared temperature (20°C), the temperature effects should also be further determined.

A slow elimination was observed for enrofloxacin following single oral administration with an elimination half-life of 129.44 h in plasma. The present plasma T_1/2λ*Z*_ value was close to those reported in rainbow trout [115–166 h; (17)]; however, longer than those reported in tilapia [19.36 h; (14)], red pacu [28.9 h; (29)], and crucian carp [64.66–73.7 h; (12)]. These differences indicated that enrofloxacin elimination was slower in Yellow River carp. We do not know the real reasons for these differences, but the species and breeding environment changes may be reasonable.

After single oral administration, the elimination rate constants ranged from 0.0024 to 0.008 h^−1^ in different tissues, indicating slow elimination of enrofloxacin in Yellow River carp. The T_1/2λ*Z*_ values also varied greatly among tissues. The longest *T*_1/2λ*Z*_ (291.13 h) was observed in the gill, which was 3.4 times the shortest value in the bile (86.22 h; Table 2), indicating the elimination of enrofloxacin was slowest from gill and fastest from bile. However, the quickest elimination of enrofloxacin from the gill has been reported in northern snakeheads with the *T*_1/2λ*Z*_ value of 68.7 h following multiple oral doses (18). The current *T*_1/2λ*Z*_ values of enrofloxacin were significantly longer than those reported in grass carp [42.4, 42.7, and 86.3 h in the kidney, serum, and liver, respectively; (43)]. Shorter T_1/2λ*Z*_ values have also been reported in rainbow trout, with the values ranging from 36.48 to 71.88 h in different tissues (19). However, longer muscle plus skin *T*_1/2λ*Z*_ value (115.14 h) has been reported in largemouth bass (22). In the present study, it should be noted that only 2–4 samples, including plasma, kidney, liver, and skin-muscle, were quantified at the last quantifiable time point (360 or 480 h), and those samples with enrofloxacin concentrations below the LOQ were omitted and not used to calculate *T*_1/2λ*Z*_. Therefore, the true average concentration is likely lower than the currently reported average one, which would impact the estimation of *T*_1/2λ*Z*_ as well as the withdrawal period.

In the present study, the highest AUC value was determined in bile (597.07 h·μg·g^−1^) followed by the gut (255.91 h·μg·g^−1^), liver (220.31 h·μg·g^−1^), kidney (216.18 h·μg·g^−1^), skin-muscle (90.99 h·μg·g^−1^), gill (85.19 h·μg·g^−1^), and plasma (67.45 h·μg·mL^−1^). The AUC ratios of tissues to plasma in bile, gut, liver, kidney, skin-muscle, and gill were 8.85, 3.79, 3.27, 3.21, 1.35, and 1.26, respectively.

After one single oral administration of enrofloxacin, the longest MRT (159.77 h) was determined in gill, followed by liver (147.37 h), plasma (111.56 h), skin-muscle (104.91 h), kidney (89.81 h), bile (80.87 h), and gut (62.89 h). The MRT values reported here were longer than those reported in rainbow trout, ranging from 53.40 h in the gut to 104.4 h in the skin (19). And the current MRT values in plasma and skin-muscle were also longer than those in northern snakehead (62.64 and 84.58 h, respectively); the current values in the liver and gill were longer than those in the northern snakehead (134.62 and 98.61 h, respectively) (18). These inconsistencies may be related to differences in species and breeding environment. The area under the first-moment curve (AUMC_0−∞_) values were also calculated for enrofloxacin in different tissues, and they were in the following order: bile > liver > kidney > gut > gill > skin-muscle > plasma.

After single oral administration, multiple peaking profiles were observed for enrofloxacin in all tested tissues concentration-time curves. Similar results have also been reported in turbot (34), Atlantic salmon (32), red pacu (29), *Takifugu flavidus* (30), Koi carp (42), and crucian carp (39). It was shown in Figure 1B that the last peak at 96 h was significantly higher than those previous ones in the bile concentration-time curve. Additionally, the concentration of enrofloxacin in bile at most time points was higher than the corresponding concentration in plasma, which indicated high transportability from plasma to bile. Therefore, the excretion with bile might be the primary elimination route of enrofloxacin in Yellow River carp. Only the gut tissues were collected in the present study, not the intestinal contents. Therefore, we were not able to correlate bile excretion with intestinal absorption. And enterohepatic recirculation might be the reason for the multiple peaking profiles. In a previous study conducted in silver crucian carp (33), enterohepatic circulation was also speculated to explain multiple peaking for enrofloxacin. Similarly, enterohepatic circulation has also been reported for flumequine in channel catfish (44). However, this should be further validated in Yellow River carp.

As concentration-dependent antibacterials, the PK/PD indices for fluoroquinolones have been proved as plasma AUC/MIC ratio ≥125 and C_max_/MIC ratio ≥10 to provide clinical and bacteriological success and prevent the emergence of resistance (45). As recommended by VetCAST (46), an epidemiological cut-off value (ECOFF) should be used as a surrogate when the clinical breakpoint (CBP) for antimicrobial drugs is not available. To our knowledge, neither CBP nor ECOFF of enrofloxacin against *A. hydrophila* and *A. sobria* was available in Yellow River carp. However, its MIC50 was recently reported as 0.25 and 0.5 μg/mL against *A. hydrophila* and *A. sobria*, respectively (10). And its MIC50 and MIC90 values were also observed as 0.016 and 0.125 μg/ml against *F. columnare*, respectively (47). However, another study determined its ECOFF against *F. columnare* as 0.03 μg/ml (48). All these MIC values were old [even from 1987; (47)] and not from the Yellow River carp. Assuming enrofloxacin has the same MIC50 values, the calculated AUC/MIC ratios against *A. sobria, A. hydrophila*, and *F. columnare* are 134.9, 269.8, and 4215.625, respectively; and the corresponding C_max_/MIC ratios were 1.58, 3.16, and 49.375, respectively. Therefore, the current 10 mg/kg enrofloxacin oral administration might provide suitable plasma concentrations to inhibit *F. columnare, A. sobria*, and *A. hydrophila*. The appropriate use of antimicrobial drugs in aquaculture depends on rapid bacterial diagnosis and accurate antimicrobial susceptibility testing. The abundant and accurate aquaculture species-specific MIC data could successfully predict the clinical outcome of antimicrobial treatment. Therefore, the enrofloxacin MIC data against these three Yellow River carp-specific pathogens should be further collected to validate the dosing regimen.

Although only a single dose of enrofloxacin was orally administered to Yellow River carp, the withdrawal period was calculated as 379.2 °C-day. Given the temperature effects on drug elimination and the current long half-lives in different tissues, the depletion study following multiple oral doses should be carried out in Yellow River carp reared at some lower temperatures.

## Conclusion

The present results showed that enrofloxacin had a wide distribution in different tissues, however slow absorption and elimination in Yellow River carp. Only trace levels of ciprofloxacin were observed occasionally in liver samples; however, enrofloxacin exhibited large distribution in bile, indicating that bile excretion might be the primary elimination route of enrofloxacin in Yellow River carp. Based on the calculated PK/PD indices of AUC/MIC or C_max_/MIC, the current enrofloxacin dosing regimen might have a positive therapeutic effect on the infection of *F. columnare, A. sobria*, or *A. hydrophila*. However, the depletion study following multiple oral doses should be carried out in Yellow River carp reared at some lower temperatures, and the withdrawal period should be further calculated.

## Data Availability Statement

The raw data supporting the conclusions of this article will be made available by the authors, without undue reservation.

## Ethics Statement

The animal study was reviewed and approved by the Institutional Animal Care and Use Committee (IACUC) of Henan University of Science and Technology.

## Author Contributions

FanY conceived this project. C-SZ, Z-WS, and M-HD performed the pharmacokinetics and tissue distribution experiments. HW, H-TS, and K-LM determined the enrofloxacin concentrations in collected samples. FanY and FangY performed the pharmacokinetic analysis. FanY wrote this manuscript with support from C-SZ. All authors read and approved this final manuscript.

## Funding

This research was funded by the Natural Science Foundation of Henan (Grant No. 212300410037) and the National Natural Science Foundation of China (Grant No. 31402253).

## Conflict of Interest

The authors declare that the research was conducted in the absence of any commercial or financial relationships that could be construed as a potential conflict of interest.

## Publisher's Note

All claims expressed in this article are solely those of the authors and do not necessarily represent those of their affiliated organizations, or those of the publisher, the editors and the reviewers. Any product that may be evaluated in this article, or claim that may be made by its manufacturer, is not guaranteed or endorsed by the publisher.
