# Supplementary material for: Pharmacokinetics and Tissue Distribution of Enrofloxacin Following Single Oral Administration in Yellow River Carp (Cyprinus carpio haematoperus)
Source: Front Vet Sci. 2022 Feb 4;9:822032. doi: 10.3389/fvets.2022.822032 (PMC8855120; doi:10.3389/fvets.2022.822032)
Supplement: Supplementary file 1 [file Table_1.docx]

**Pharmacokinetics and tissue distribution of enrofloxacin following single oral administration in Yellow River carp (*Cyprinus carpio haematoperus*)**

Fan Yang*, Chao-Shuo Zhang, Ming-Hui Duan, Han Wang, Zhe-Wen Song, Hao-Tian Shao, Kai-Li Ma, Fang Yang

College of Animal Science and Technology, Henan University of Science and Technology, Luoyang 471023, P.R. China

**Supplementary Tables**

Table S1. The values of some parameters used to estimate the goodness of fit statistic for the terminal elimination phase

| Parameters | Unit | plasma | bile | skin-muscle | liver | kidney | gill | gut |
| --- | --- | --- | --- | --- | --- | --- | --- | --- |
| Rsq | unitless | 0.988 | 0.962 | 0.993 | 0.904 | 0.958 | 0.897 | 0.909 |
| Rsq_adjusted | unitless | 0.975 | 0.942 | 0.989 | 0.809 | 0.916 | 0.795 | 0.886 |
| Corr_XY | unitless | -0.994 | -0.981 | -0.996 | -0.951 | -0.979 | -0.947 | -0.953 |
| No_points_lambda_z | unitless | 3 | 4 | 4 | 3 | 3 | 3 | 6 |

Rsq: Goodness of fit statistic for the terminal elimination phase; Rsq_adjusted: Goodness of fit statistic for the terminal elimination phase, adjusted for the number of points used in the estimation of λ_Z_; Corr_XY: Correlation between time (X) and log concentration (Y) for the points used in the estimation of λ_Z_; No_points_lambda_z: Number of points used in computing λ_Z_.
